# Enhanced photocatalytic degradation of Acid Blue dye using CdS/TiO_2_ nanocomposite

**DOI:** 10.1038/s41598-022-09479-0

**Published:** 2022-04-06

**Authors:** Nida Qutub, Preeti Singh, Suhail Sabir, Suresh Sagadevan, Won-Chun Oh

**Affiliations:** 1 Department of Chemistry, Jamia Millia Islamia, New Delhi, 110025 India; 2 Department of Fibers and Textile Processing Technology, Institute of Chemical Technology, Mumbai, 400019 India; 3 Department of Chemistry, Aligarh Muslim University, Aligarh, 202002 India; 4 Nanotechnology & Catalysis Research Centre, University of Malaya, 50603 Kuala Lumpur, Malaysia; 5 Department of Advanced Materials Science and Engineering, Hanseo University, Seosan-si 356-706, Chungnam, Korea

**Keywords:** Environmental sciences, Chemistry, Materials science, Nanoscience and technology

## Abstract

Photocatalytic degradation is essential for the successful removal of organic contaminants from wastewater, which is important for ecological and environmental safety. The advanced oxidation process of photocatalysis has become a hot topic in recent years for the remediation of water. Cadmium sulphide (CdS) nanostructures doped with Titanium oxide (CdS/TiO_2_) nanocomposites has manufactured under ambient conditions using a simple and modified Chemical Precipitation technique. The nanocomposites crystal structure, thermal stability, recombination of photo-generated charge carriers, bandgap, surface morphology, particle size, molar ratio, and charge transfer properties are determined. The production of nanocomposites (CdS-TiO_2_) and their efficient photocatalytic capabilities are observed. The goal of the experiment is to improve the photocatalytic efficiency of TiO_2_ in the visible region by doping CdS nanocomposites. The results showed that as-prepared CdS-TiO_2_ nanocomposites has exhibited the highest photocatalytic activity in the process of photocatalytic degradation of AB-29 dye, and its degradation efficiency is 84%. After 1 h 30 min of visible light irradiation, while CdS and TiO_2_ showed only 68% and 09%, respectively. The observed decolorization rate of AB-29 is also higher in the case of CdS-TiO_2_ photocatalyst ~ 5.8 × 10^−4^mol L^−1^ min^−1^) as compared to the reported decolorization rate of CdS ~ 4.5 × 10^−4^mol L^−1^ min^−1^ and TiO_2_ ~ 0.67 × 10^−4^mol L^−1^ min^−1^. This increased photocatalytic effectiveness of CdS-TiO_2_ has been accomplished by reduced charge carrier recombination as a result of improved charge separation and extension of TiO_2_ in response to visible light.

## Introduction

Wastewater recycling by photocatalysts could be a solution to many countries' long-standing freshwater shortages^1^. Water pollution-related environmental issues have been a serious issue in recent decades, negatively affecting human health and the ecology. Access to safe drinking water has risen to the top of the priority list for long-term economic growth and societal well-being. Water is necessary for life and a valuable resource for civilization; it also plays an important role in natural ecosystems and climate regulation. Water stress is largely a problem of scarcity of water, but it can also be caused by deterioration of water quality and a lack of proper water management^2^. Wastewater from the pulp, leather, fabric, polymer, pesticide and pharmaceutical sectors comprises a variety of organic contaminants, including dyes, that contaminate water and harmful life on the planet^3–5^. Separating and degrading these organic waste products has taken precedence in ensuring a nontoxic and pollution-free environment^6^. Photocatalysis an advanced oxidation process (AOP) is currently being utilized to remove colours from water without producing any hazardous by-products^6^. Fujishima and Honda^7^ established the theory of photocatalysis, which focuses on the generation of highly reactive intermediates, such as hydroxyl radicals (**·**OH), which rapidly oxidize a wide range of pollutants. The photocatalyst’s catalyst activity is highly dependent on the light sources; ultraviolet (UV) radiations have also been used^8–10^. A photocatalyst is a compound that facilitates the acceleration and enhancement of a light-induced reaction without being consumed. A photocatalyst turns solar energy into chemical energy, which can be utilized for pollution and sustainable applications such as water purification, environmental remediation, self-cleaning surfaces, hydrogen synthesis via water cleavage, and CO_2_ conversion to hydrocarbon fuels. Solar energy is the purest and most abundant source of radiating energy that does not include any contaminants^11^.

In recent times, lot of interest in the development of novel photocatalytic technologies for a wide range of environmental applications, such as water remediation, oil spill and another pollution cleaning. Some energy-related research has focused on the utilization of photocatalysts for fuel production (e.g., H_2_), specifically through water splitting^12^. Solar radiation is used to convert pollutants from complicated molecules to simple and nontoxic compounds, avoiding the need for additional treatment, disposal, or the use of expensive oxidizing chemicals. Titanium oxide nanoparticle (TiO_2_ NP) is widely used as a photocatalyst in the field of advanced oxidation technology due to its advantageous properties, such as exceptional optical and electronic properties, strong oxidative power, high photocatalytic efficiency, high stability, robust physical and chemical stability, low cost, low toxicity, stability in aqueous solutions, and eco-friendliness^13,14^. It is a well-known potent photocatalyst for degrading a wide range of molecules^15^, including inorganic and organic compounds^16,17^. Titanium oxide (TiO_2_) photocatalytic activity is influenced not only by its bulk energy band structure, but also by the crystalline structure, textural characteristics, surface area, and particle size of the TiO_2_ powder^18^. The photocatalytic activity of TiO_2_ can be improved by changing the physicochemical nature of the nanoparticle surface to increase the adsorption of harmful compounds to be destroyed and reduce the likelihood of charge carrier recombination^19,20^.

In the previous two decades, there has been substantial progress in the creation of effective photocatalytic materials, with a high number of research articles released each year. Advances in nanotechnology have substantially connected with improvements in the performance of photocatalytic materials. Titanium dioxide has been extensively explored for photocatalysis because it has numerous advantages such as strong photocatalytic activity, great physical and chemical stability, low cost, non-corrosive, non-toxicity, and high availability^21,22^. When TiO_2_ NP absorbs light at 385 nm, electrons (e^−^) are excited from the valence band to the conduction band, leaving a positively charged vacancy known as a hole (h^+^)^23^. The hole is a strong potent oxidizing agent in and of itself, and in the presence of water and molecular oxygen, it can generate hydroxyl radicals or directly oxidize adsorbed molecules on the NP surface. TiO_2_ has the advantage of being able to execute functions while using sunlight as a source of energy. Due to charge recombination, these photoinduced charges have a relatively brief lifetime, releasing the absorbed light energy as heat with no chemical consequence. These charges can also migrate to lower-energy trap sites, where they can still recombine or participate in redox processes with adsorbed species. As a result, preventing electron–hole recombination is critical for improving TiO_2_ efficiency. Furthermore, TiO_2_ can only absorb in the UV area of the solar spectrum, which accounts for just about 5% of total solar energy falling on the earth's surface^24^, restricting its application. As a result, numerous attempts have been made to increase charge separation by altering the surface or bulk properties of TiO_2_, such as doping^25^, metal deposition^26^, size reduction, and coupling of two semiconductors, consequently boosting photocatalytic activity^27,28^. The photo-response of TiO_2_ can be improved by combining it with a semiconductor that has a smaller bandgap and a higher conduction band than TiO_2_. To begin with, a narrow bandgap will boost solar energy absorption efficiency by pushing it into the visible range. Second, photogenerated electrons in the narrow bandgap SC's conduction band (CB) will be injected into TiO_2_'s CB, reducing charge recombination and keeping oxidation and reduction processes in different reaction sites^29^. TiO_2_ has a bandgap of 3.2 eV for the anatase phase and 3.0 eV for the rutile phase, respectively. As a result, under visible light irradiation, TiO_2_ is nearly inert, making it unable to use solar energy sustainably^30^.

On the other hand, TiO_2_ has a number of disadvantages that limit its use in photocatalysis. To begin with, photogenerated electrons and holes coexist in the titania particle, with a high likelihood of recombination. As a result, the desired chemical changes occur at modest rates in relation to the absorbed light energy. Because of the very large bandgap energy (3.2 eV), photoactivation requires ultraviolet light, resulting in very low efficiency in utilizing solar radiation. When compared to visible light, UV light makes up just around 5% of the solar spectrum (45% only)^31–33^. Furthermore, due to its non-porous nature and polar surface, titania has a poor absorption capacity for non-polar organic contaminants^34^. The recovery of nano-sized titania particles from treated water is also a challenge, both economically and in terms of safety^35^. Agglomeration and aggregation alter the photoactivity and light absorption of TiO_2_ nanoparticles^36–38^. To alleviate these limitations, several techniques have been proposed in the open literature. These strategies aim to increase the utilization of solar energy by extending the wavelength of photoactivation of TiO_2_ into the visible region of the spectrum; preventing electron/hole pair recombination and thus allowing more charge carriers to diffuse to the surface; increasing the adsorption affinity of TiO_2_ towards organic pollutants, and preventing aggregation and agglomeration of nano-titania particles while easing their recovery. Many efforts have been made to improve TiO_2_ NPs immobilised on TiO_2_ nanofibers (TNF), making them a promising platform for photocatalytic wastewater treatment and other applications such as sensing, photovoltaics, and photocatalytic water splitting^39–41^. Furthermore, recent advances in the design and engineering of metal oxide–graphene–noble metal-based high-performance photocatalyst systems are developed, with an emphasis on the associated mechanisms and their applications in various photocatalytic processes^42,43^. Several reviews on the development of ways to overcome the limits of titania photocatalysis have been published in recent years^44–47^. Ion doping, for example, anatase–rutile phase coexisting^48^, ion doping^49^, or p-n heterojunction formation^50^. Many elements were doped into TiO_2_, such as Ag^51^, Fe^52^, N^53,54^, S^55^, C^56^ and B^57^, etc. Doping ions can introduce new energy levels into TiO_2_, lowering the bandgap energy and increasing photocatalytic activity, especially when exposed to visible light. Consequently, the development of charge trapping sites by foreign ions can effectively lower the rate of electron–hole recombination^58^.

On the other hand, Cadmium sulphide (CdS) is an excellent photocatalyst in the visible region but has low quantum efficiency due to low stability in solution due to Cd^2+^ ion leaching. As a result, despite its relatively high photoactivity, significant efforts are being made to increase photocatalytic stability. Attempts to improve the photocatalytic efficiency of CdS have included changing the surface structure of CdS NPs by controlling morphology, doping transition metal ions into CdS, depositing CdS to Nafion membranes, graphene sheets, or carbon nanotubes to obtain a uniform, homogeneously distributed CdS QDs, and coupling CdS with another semiconductor. Combining different bandgap semiconductors to make solid solutions is an effective approach to adjust the potential of conduction and valence bands by making successive composition changes^59^. Due to the suitable band gap (2.4 eV) of CdS^60^, lower CB than TiO_2_, great optical property, and possible application of CdS in photo-electrochemistry, photocatalysis, and water splitting systems, coupling of TiO_2_ with cadmium sulphite (CdS) has been widely researched^61^. Due to its low bandgap (2.4 eV), which enables its visible light response, CdS is the most important chalcogenides semiconductor as a hydrogen production catalyst^62,63^. The restricted separation efficiency of photogenerated charge carriers can overcome either by employing CdS in the form of QDs due to a shorter transit path or by integrating CdS onto support materials, such as TiO_2_^64,65^.

CdS doped TiO_2_ nanotube composites were previously synthesized by chemical bath deposition, and their light-harvesting performance was 2.9 times than that of pure TiO_2_ nanotubes. Under UV light irradiation, the CdS doped TiO_2_ nanotube composite had better photocatalytic activity and photodegradation efficiency than pure TiO_2_ nanotube and the degradation efficiency of methyl orange was about 42 percent at a UV intensity of 32 W^66^. Rao et al.^67^ created CdS/TiO_2_ core/shell nanorods with variable shell thickness to reduce charge carrier recombination and photo corrosion when exposed to UV–Vis light. Du et al. created the same type of composite, but with different morphology, by fabricating pyramid-like CdS nanoparticles and growing them on porous TiO_2_. The H_2_ generation rate of 5 mol% CdS-TiO_2_ was 1048.7 mol h^−1^ g^−1^ under UV–Vis irradiation and without noble-metal co-catalysts, which is about six times and 1.5 times greater than pure TiO_2_ and CdS, respectively^68^.

In the present study, a series of TiO_2_ NPs were synthesized in this study to investigate the effect of reactant concentration on size, shape, crystal structure, thermal, optical, and photocatalytic activities. According to prior research, a number of manufactured CdS NPs were used in the photocatalytic experiment, and it was discovered that the CdS NPs, has an excellent photo-response, but not suitable for photocatalytic water purification. TiO_2_ is not photoactive under the visible region of the solar spectrum. Thus, in order to make CdS potentially applicable for water purification and utilization of TiO_2_ in the visible region a nanocomposite of cadmium sulphide and titanium dioxide (CdS-TiO_2_) was also synthesized and the effect of CdS on TiO_2_ and vice versa was studied.

## Materials and methods

### Synthesis of titanium oxide nanoparticle (TiO_2_ NP) by precipitation technique

Controlled precipitation of nanoparticles from precursors dissolved in a solution was used to make TiO_2_ NP. The reductive hydrolysis of Titanium Tetra Isopropoxide (TTIP) in methanol at ambient temperature and pressure without calcinations was proposed for the manufacture of TiO_2_ nanoparticles^69^. By adjusting the concentration of TTIP while maintaining the amount of methanol (24.44 M) constant at 100 mL, a series of TiO_2_ NP (Tma, Tmb, Tmc, Tmd, and Tme) was synthesized. Tma = 0.25 M, Tmb = 0.20 M, Tmc = 0.15 M, Tmd = 0.1 M, and Tme = 0.05 M have different TTIP concentrations. The reactions were carried out as follows: 100 mL methanol (24.44 M) was placed in a conical flask, and TTIP was added dropwise (20 drops per minute) while vigorous stirring continued for another 5 h. White precipitates observed were washed with water and acetone several times and then air-dried. The crystal structure of the produced TiO_2_ was expected to be a mix of anatase, brookite, and rutile.

### Chemical route for the synthesis of cadmium sulphite doped titanium oxide (CdS-TiO_2_)

In a reaction vessel, 100 mL aqueous Cd (NO_3_)_2_ (0.085 M) was added dropwise with continuous stirring, followed by 50 mL methanol (24.44 M). The reaction was then carried out for 1 min in the H_2_S environment with vigorous stirring and then continued for another 2 h. The colour of the solution changed from clear to yellow. 3.53 mL TTIP (0.1 M) was added drop-by-drop to this solution (20 drops per minute). The stirring was extended for another 5 h. The hue of the solution had changed to a faint yellow.

### Characterization techniques

The synthesized TiO_2_ NP and CdS-TiO_2_ NC were characterized by elemental, structural, optical and thermal techniques. Elemental analysis and chemical compositions were examined by energy-dispersive X-ray spectroscopy (EDS, JEOL, JSM6510LV) and Fourier Transform Infrared Spectroscopy. The structural properties were analysed by employing powder X-ray diffraction (Miniflex-TM II Benchtop, Rigaku Co-operation, Tokyo, Japan). Surface morphology and size was characterized by Scanning Electron Microscopy (JEOL, JSM6510LV) and Transmission Electron Microscopy (JEOL, JEM2100). Thermal properties were determined by Thermal Gravimetric Analysis (TGA). The optical properties were determined by employing UV–Visible Spectroscopy (Shimadzu UV-1601).

### Photocatalytic experiment

The decolorization of a dye derivative Acid Blue-29 (AB-29) in the presence of UV light was used to investigate the photocatalytic activity of TiO_2_ nanoparticles. The photocatalytic studies were carried out in an immersion well Pyrex glass photoreactor (inner and outer jacket) with a magnetic bar, water circulating jacket, and a molecular oxygen opening. A 125 W medium pressure mercury lamp was used to irradiate the area (Philips). The optimal catalyst dosage was established by irradiating the dye (AB-29) aqueous solution with various strengths of TiO_2_ nanoparticles. 180 mL dye (AB-29) solution (0.06 mM) containing manufactured nanocatalysts (1 g L^−1^) were magnetically swirled in the dark for at least 20 min in the presence of ambient oxygen to achieve dye (AB-29) and nanocatalyst surface adsorption–desorption equilibrium. After reaching equilibrium, the first aliquot (5 mL, 0 min) was removed and the irradiation process began. During the irradiation, other aliquots of 5 mL were taken at regular intervals and examined following centrifugation. Changes in absorption were used to track the decolorization of AB-29 using a UV–Visible spectroscopic analysis approach (Shimadzu UV–Vis 1601). The dye concentration was determined using a standard calibration curve based on the dye's absorbance at various known values. The photocatalytic activity of the CdS-TiO_2_ nanocomposite was tested by examining the decolorization of AB-29 in the presence of visible light using a halogen linear lamp (500 W, 9500Lumens) as a light source and comparable experimental conditions as described before. Photocatalytic studies were conducted for five cycles using the same batch of nanomaterial photocatalysts to determine the reusability and recyclability of the nanomaterials as catalysts. Before each photocatalytic run, the nanocomposite catalyst was rinsed with double distilled water after each cycle and a fresh solution of AB-29 was added.

## Results and discussion

### Spectroscopy analysis

**Figure 1:**
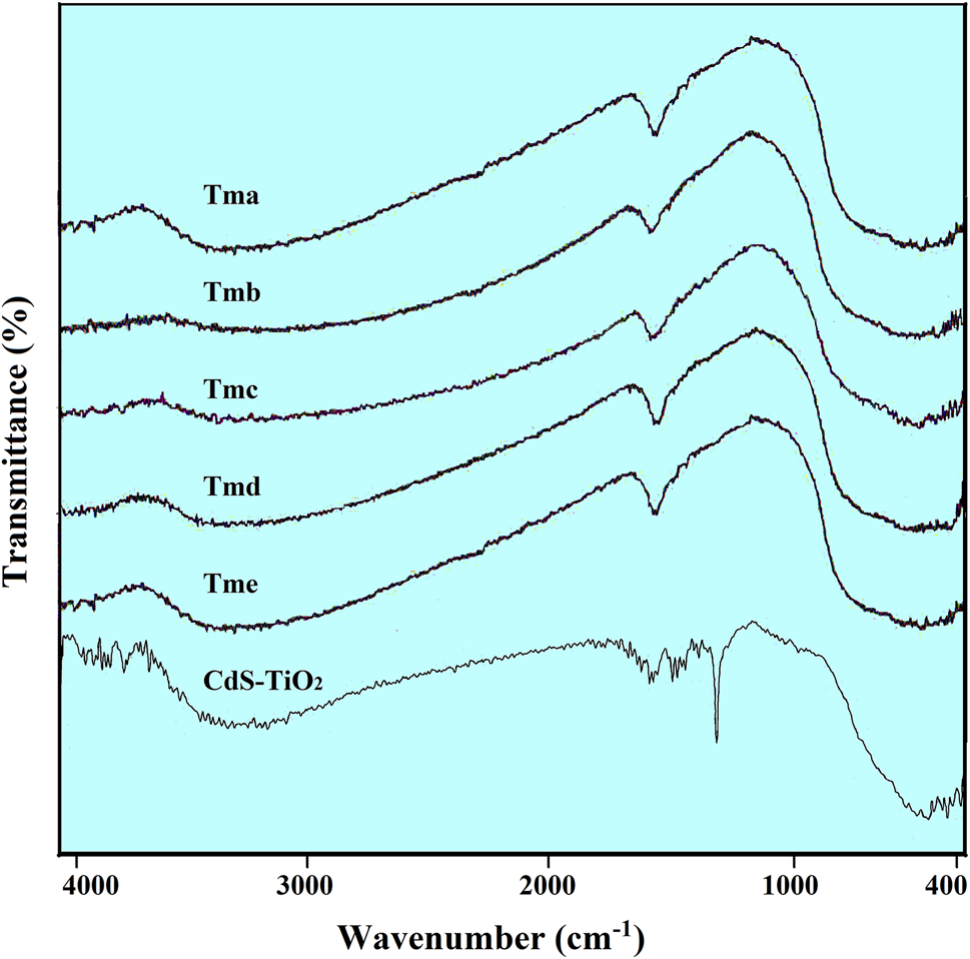
The FTIR Spectra of the synthesized TiO_2_ nanoparticles (Tma, Tmb, Tmc, Tmd and Tme) and its nanocomposites with CdS (CdS-TiO_2_).

The purity and composition of the samples were validated by FTIR spectra as shown in Fig. 1, which revealed multiple peaks related to TiO_2_ in all samples without any other elemental contamination. In the 800–400 cm^−1^ area, bands for Ti–O and Ti–O–Ti bands were detected. The FTIR spectra of TiO_2_ could be in the form of a broad band centred at 400–800 cm^−1^ due to the Ti–O bond vibration in the TiO_2_ lattice^70^ or peaks centred at 760 cm^−1^, 680 cm^−1^, 600 cm^−1^, 560 cm^−1^, 500 cm^−1^, 468 cm^−1^, 410 cm^−1^, 385 cm^−1^ and 350 cm^−1^ attributable to^71,72^. The FTIR spectra of TiO_2_ nanoparticles (Tma, Tmb, Tmc, Tmd, and Tme) and their nanocomposites with CdS are shown (CdS-TiO_2_) in Fig. 1. The varied peaks generated by TiO_2_ nanoparticles and their nanocomposites with CdS nanoparticles are explained in Table 1^73,74^.

**Table 1:** Significance of peaks obtained in the FTIR spectra of the synthesized TiO_2_ nanoparticles (Tma, Tmb, Tmc, Tmd and Tme) and CdS-TiO_2_.

Peak	Range	Intensity	Significance
A	(400–420)	Small and weak	Cd-S bond (CdS nanoparticles)
B	(400–800)	broadband	Ti–O bond vibration (TiO_2_ nanoparticles)
C	(570–620)	Small and weak	S–S bond (crystal S–S bond)
D	(1380–1420)	Sharp or Broad	C–H bending of CH_3_ (Acetone)
E	(1620–1740)	Small and weak	CO_2_ bending or C-H bending (Acetone)
F	(3140–3470)	Broad	Intermolecular H-bonds (Lattice water)

The FTIR spectra of TiO_2_ NPs (Tma, Tmb, Tmc, Tmd, and Tme) and CdS-TiO_2_ NC in this study were in the shape of a large peak in the 400–800 cm^−1^ region with multiple tiny peaks. Stretching Vibrations of hydroxyl (OH) groups of water adsorbed by the samples were ascribed to the broad peak showing at 3100–3600 cm^−1^. Such TiO_2_–OH groups are formed as a result of the hydrolysis reaction in the process. The bending modes of –OH groups of water molecules adsorbed on the surface of the catalyst are responsible for the peak at 1628 cm^−1^^75^. CO_2_ deposited on the surface of the particles showed a weak absorption band at 1620–1630 cm^−1^. Adsorption of water and CO_2_ was ubiquitous for all powder samples exposed to the atmosphere, and significantly more evident for nanosized particles with large surface areas, as well known^75^. In the case of CdS-TiO_2_, the existence of a broad band centred at 400–800 cm^−1^ with numerous little peaks in it proved the creation of CdS, and the presence of broadband centred at 400–800 cm^−1^ with several small peaks in it indicated the presence of TiO_2_ in the nanocomposite. The creation of a sandwich structure with CdS at the centre (core) and TiO_2_ NPs surrounding CdS as a shell result in CdS stretching at 605 cm^−1^. The FTIR spectra of the reaction product, obtained after heating the precursor at 300 °C for 2 h, reveal no absorption peak in the upper-frequency area but a sharp peak below 500 cm^−1^, which could be attributable to the Ti–O–Ti bond stretching frequency modes in titanium dioxide^76^. Individual bands at 675 cm^−1^ may be related to the anatase phase, and are attributed to Ti–O bond stretching vibration^77^. Stretching vibrations of functional groups CH, CH_2_, and CH_3_ are represented by the bands at 2963, 2907, and 2809 cm^−1^. At 1600 cm^−1^, the vibration corresponds to the C=C bond stretching vibration. The bands in the 1500–1400 cm^−1^ range are caused by bending vibrations of CH, CH_2_, and CH_3_, while those in the 1300–1200 cm^−1^ range are caused by vibrations of the methoxy group O-CH_3_ and the alcohol group C–OH^78^.

### Energy dispersive X-ray spectroscopy (EDAX) analysis

**Figure 2:**
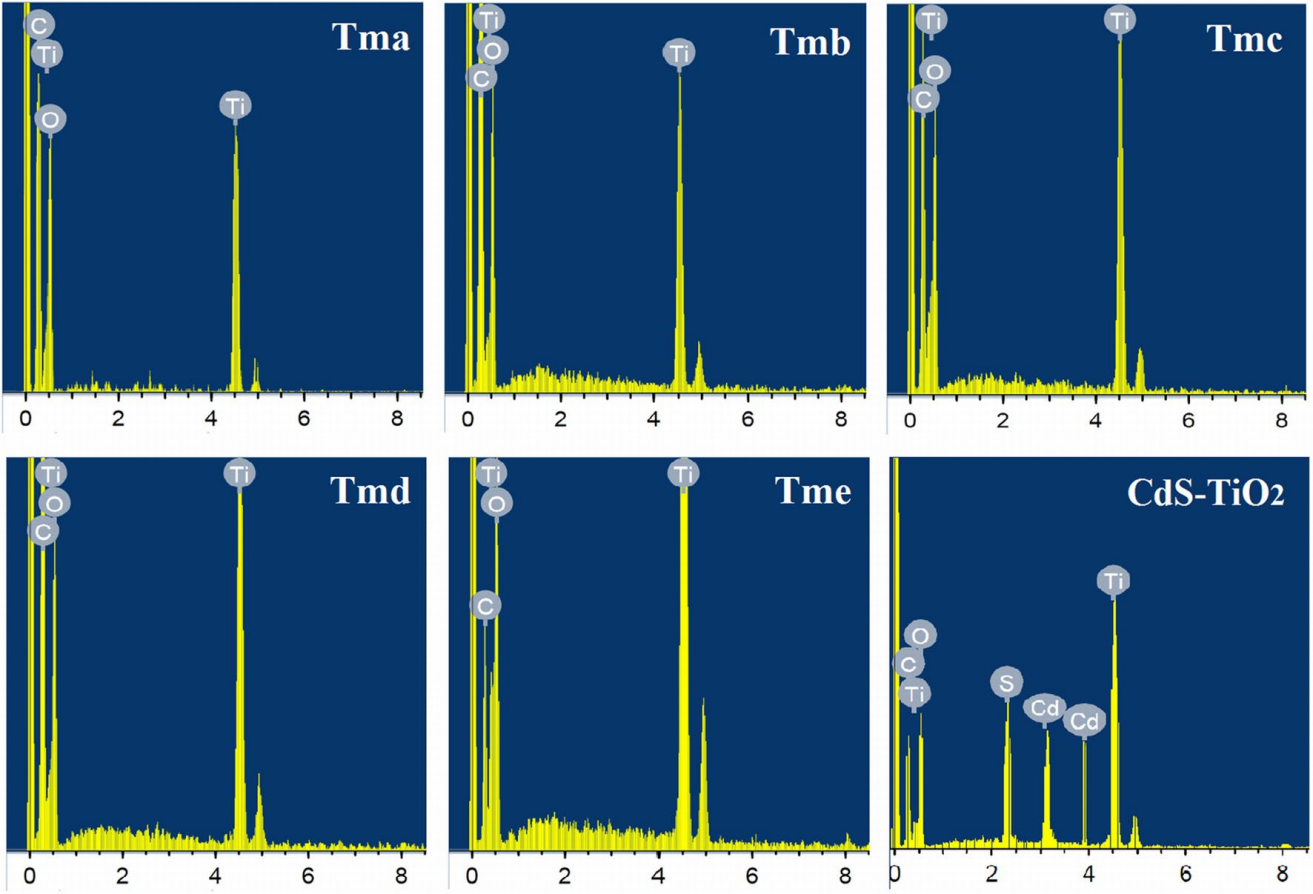
EDAX of the synthesized TiO_2_ nanoparticles (Tma, Tmb, Tmc, Tmd and Tme) and its nanocomposites with CdS (CdS-TiO_2_).

Figure 2 shows the EDAX spectra of the prepared samples. For the samples Tma, Tmb, Tmc, Tmd, and Tme, the spectra revealed the existence of Ti and O peaks, confirming the synthesis of pure TiO_2_ with no other elemental impurity. The other peaks in this graph, related to oxygen, carbon, and silicate, were caused by the sputter coating of the glass substrate on the EDS stage and not taken into account. The presence of peaks corresponding to Cd and S, as well as peaks for Ti and O, in the EDS spectra of CdS-TiO_2_ confirmed the creation of the CdS-TiO_2_ nanocomposite.

### Structural analyses

**Figure 3:**
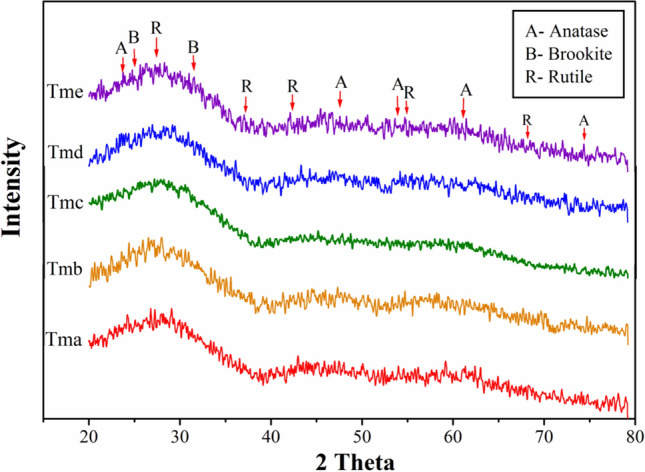
XRD patterns of synthesized TiO_2_ nanoparticles (Tma, Tmb, Tmc, Tmd and Tme).

**Figure 4:**
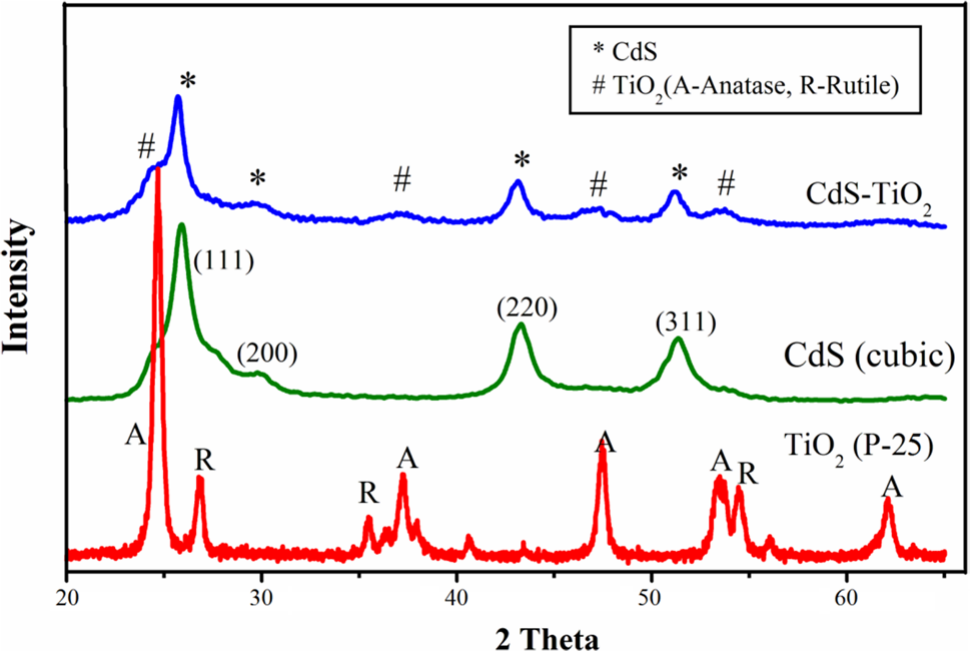
XRD pattern of CdS-TiO_2_ nanocomposites in comparison to pure cubic CdS and Degussa P-25 TiO_2_.

Diffraction and microscopic examinations were used to conduct structural evaluations. X-Ray Diffraction (XRD) Spectroscopy was used to conduct diffraction research. Due to the physical nature of the materials, the diffraction patterns for TiO_2_ NPs (Tma, Tmb, Tmc, Tmd, and Tme) were of poor quality. The XRD pattern of the produced TiO_2_ NPs (Tma, Tmb, Tmc, Tmd, and Tme) confirmed the existence of anatase, brookite, and rutile mixes (Fig. 3). TiO_2_ NPs peaks are corresponded to anatase at 2θ = 25.56° (101), 37.8° (103), 48.07° (200), 54.18° (105), 62.42° (204) and 75.2° (215), rutile at 2θ = 27.01° (110), 36.14° (101), 42.121° (111), 54.89° (211) and 68.72° (301)^79,80^ and brookite at 2θ = 30.9° (121)^49^ thus confirming the presence of mixed crystal phase. Due to the presence of mixed peaks of anatase, rutile, and brookite TiO_2_, estimating crystallite size for TiO_2_ samples (Tma, Tmb, Tmc, Tmd, and Tme) based on the X-ray diffraction peak was not achievable. The XRD pattern of CdS-TiO_2_ nanocomposites (Fig. 4) demonstrated the production of cubic CdS and anatase TiO_2_ nanoparticles. The appearance of peaks at 2θ = 25.56 (101), 37.282 (103), 48.07 (200), 54.18 (105), and 62.42 (204), which corresponded only to anatase TiO_2_, was linked to the synthesis of anatase TiO_2_ in CdS-TiO_2_^80,81^. The appearance of peaks that corresponded only to cubic CdS at 2θ = 26.719 (111), 29.900 (200), 43.000 (220), and 51.061 (311)^82,83^ verified the production of cubic CdS. The existence of mixed peaks of cubic CdS and anatase TiO_2_ made it impossible to estimate crystallite size for CdS-TiO_2_ using the X-ray diffraction peak.

### Scanning electron microscopy (SEM) analysis

**Figure 5:**
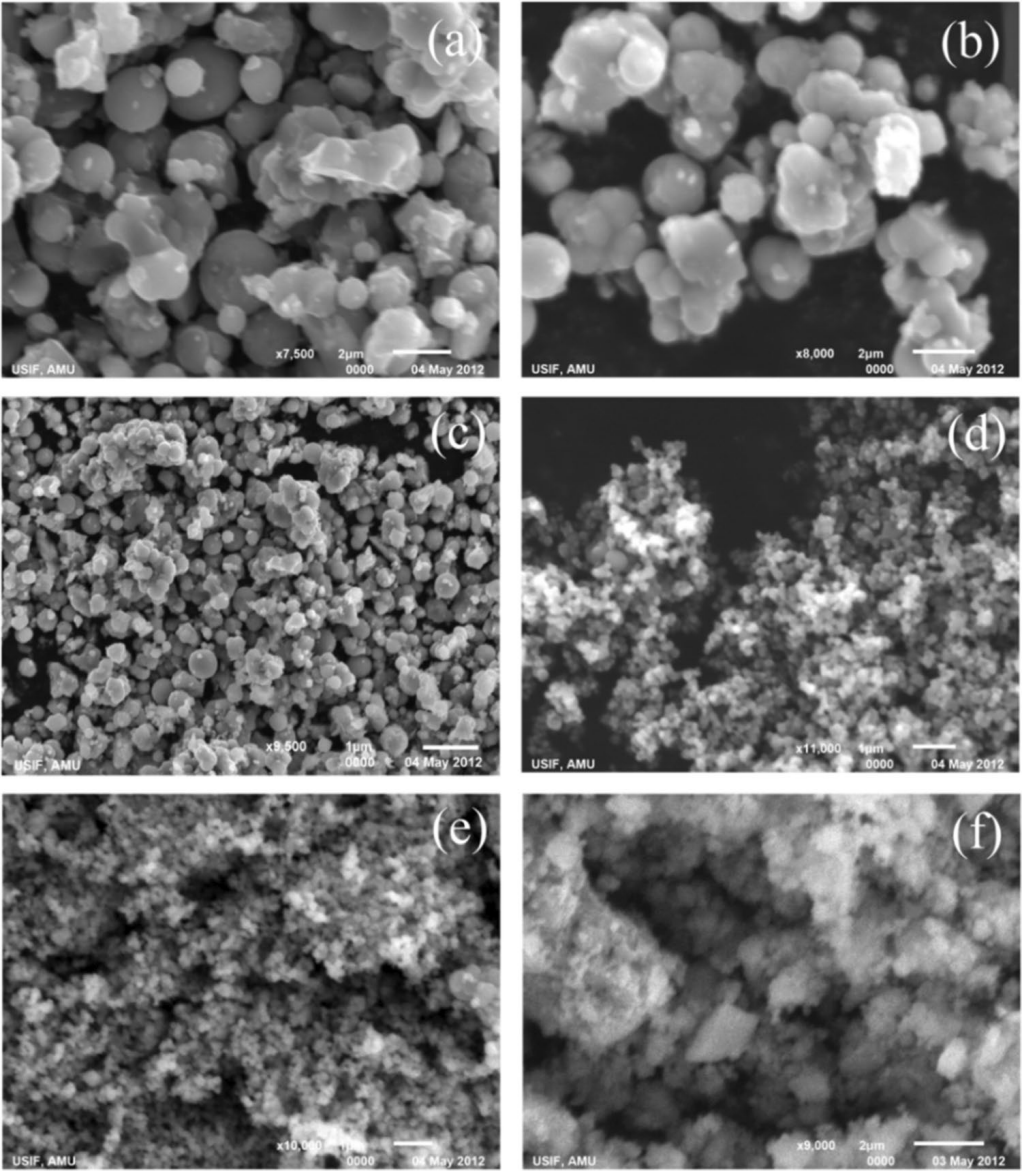
SEM images (**a**) Tma, (**b**) Tmb, (**c**) Tmc, (**d**) Tmd, (**e**) Tme and (**f**) CdS-TiO_2_.

SEM images of the synthesized TiO_2_ NP are depicted in Fig. 5a–e. The creation of well-defined spherical mesoporous TiO_2_ nanoclusters observed using SEM micrographs was attributed to the high surface energy of nanosized TiO_2_ particles^84^. By agglomerating tiny particles, pure TiO_2_ generates a layer-like structure. An amorphous mass with a very small particle structure was visible in the SEM picture of CdS-TiO_2_. Due to the thin amorphous powder, no other distinct particle shape was discernible as CdS particles uniformly covered by the TiO_2_ nanoparticles formed a sandwich-type structure. in which CdS is acting as a core surrounded by smaller TiO_2_ NPs. SEM pictures of the synthesized CdS-TiO_2_ NC are shown in Fig. 5f^85^.

### Transmission electron microscopy (TEM) analysis

**Figure 6:**
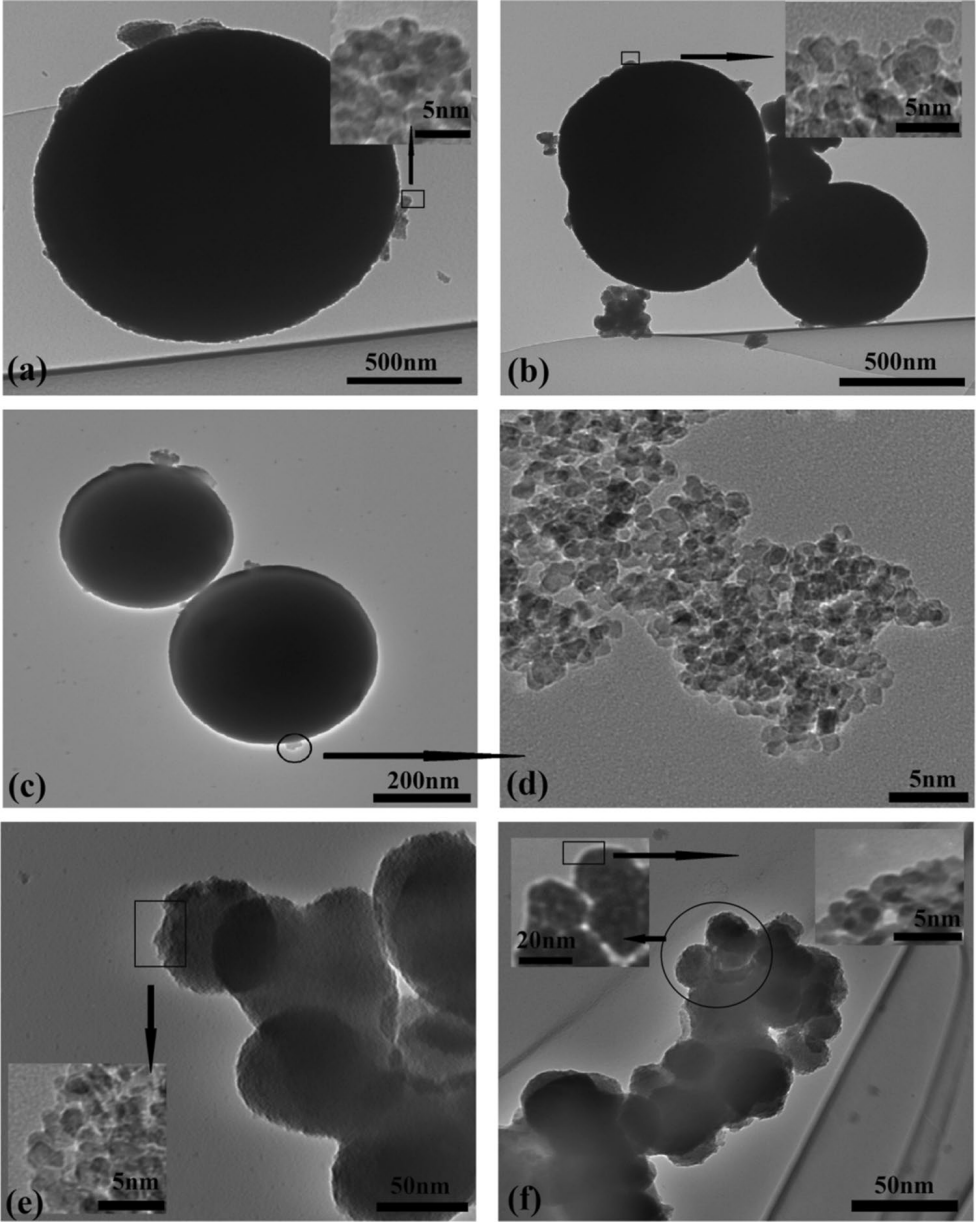
TEM images of (**a**) Tma, (**b**) Tmb, (**c**) Tmc, (**d**) magnified portion of Tmc, (**e**) Tmd, (**f**) Tme, and in the inset are the magnified portion of the corresponding images.

Figure 6a–f shows a TEM micrograph of TiO_2_ NP (Tma, Tmb, Tmc, Tmd, and Tme). The creation of spherical nanoclusters made up of very small TiO_2_ NPs (1–2 nm) can be seen in the images. The nanocluster size has shrunk from > 500 nm (Tma) to 50 nm (Tme). In all cases, however, the individual TiO_2_ nanoparticles were smaller than 2 nm.

**Figure 7:**
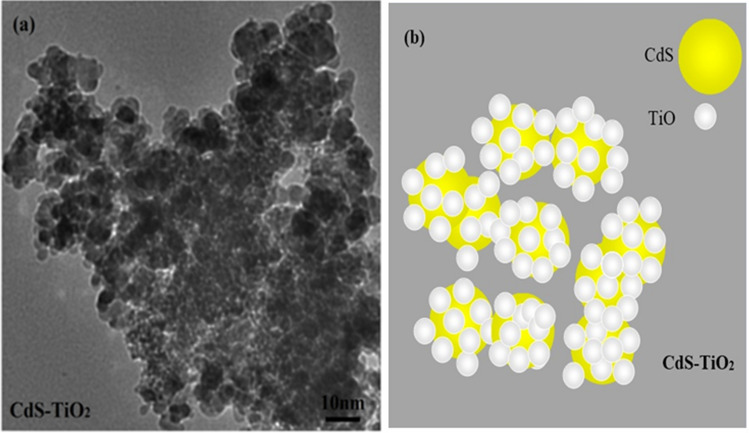
(**a**) The TEM image of the synthesized CdS-TiO_2_ nanocomposite (**b**) A representative diagram of the synthesized CdS-TiO_2_ nanocomposite.

The TEM picture of CdS-TiO_2_ (Fig. 7) verifies the presence of two different-sized nanoparticles CdS and TiO_2_ nanoparticles with close proximity to each other. TEM images of CdS-TiO_2_ (Fig. 7a) show two different-sized nanoparticles with smaller ones surrounding the larger ones in a core–shell type fashion. Further, the presence of both CdS and TiO_2_ were also confirmed by EDS and XRD test results individually. The observed two distinctly different sized particles with close proximity to each other is the direct prove of the sandwich-type model of nanoparticles with smaller particles (TiO_2_) surrounding the larger particle (CdS) like a core. Figure 7b shows a TEM image of the CdS-TiO_2_ composite. Table 2 shows the particle sizes acquired using TEM.

**Table 2:** Average particle sizes of the synthesized TiO_2_ NP (Tma, Tmb, Tmc, Tmd and Tme) and CdS-TiO_2_ NC obtained by TEM.

Nanoparticles	Tma	Tmb	Tmc	Tmd	Tme	CdS-TiO_2_
Particle size (nm)	1.5–2.0	1.5–1.7	1.2–1.5	1–1.2	0.8–1.0	5–6 (CdS), (TiO_2_)

### Thermal analyses

**Figure 8:**
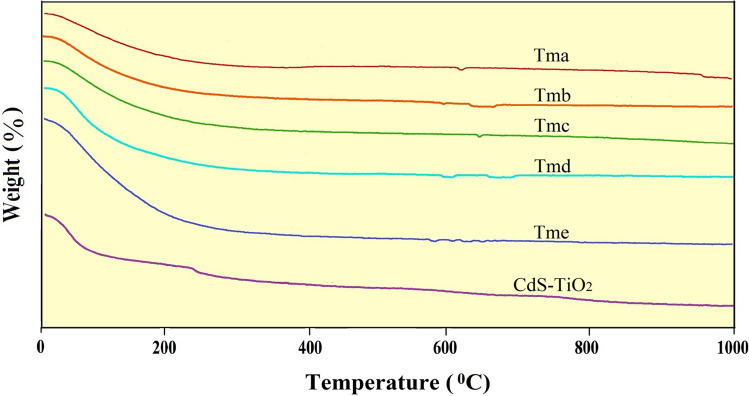
TGA graphs of synthesized TiO_2_ nanoparticles (Tma, Tmb, Tmc, Tmd and Tme) and CdS-TiO_2_ nanocomposite.

The Thermal Gravimetric Analysis (TGA) graphs were used to conduct thermal experiments. TGA findings of the synthesised TiO_2_ nanoparticles (Tma, Tmb, Tmc, Tmd, and Tme) and CdS-TiO_2_ nanocomposite are shown in Fig. 8. The TGA curve demonstrated great thermal stability, the lack of any impurity or intermediate complex, and a high melting point for the produced nanoparticles. The TiO_2_ NPs (Tma, Tmb, Tmc, Tmd, and Tme) and CdS-TiO_2_ NC were shown to be thermally stable up to 1000 °C, with a slight weight loss roughly at 100 °C, owing to the presence of moisture and other volatile solvents.

### Optical analysis

**Figure 9:**
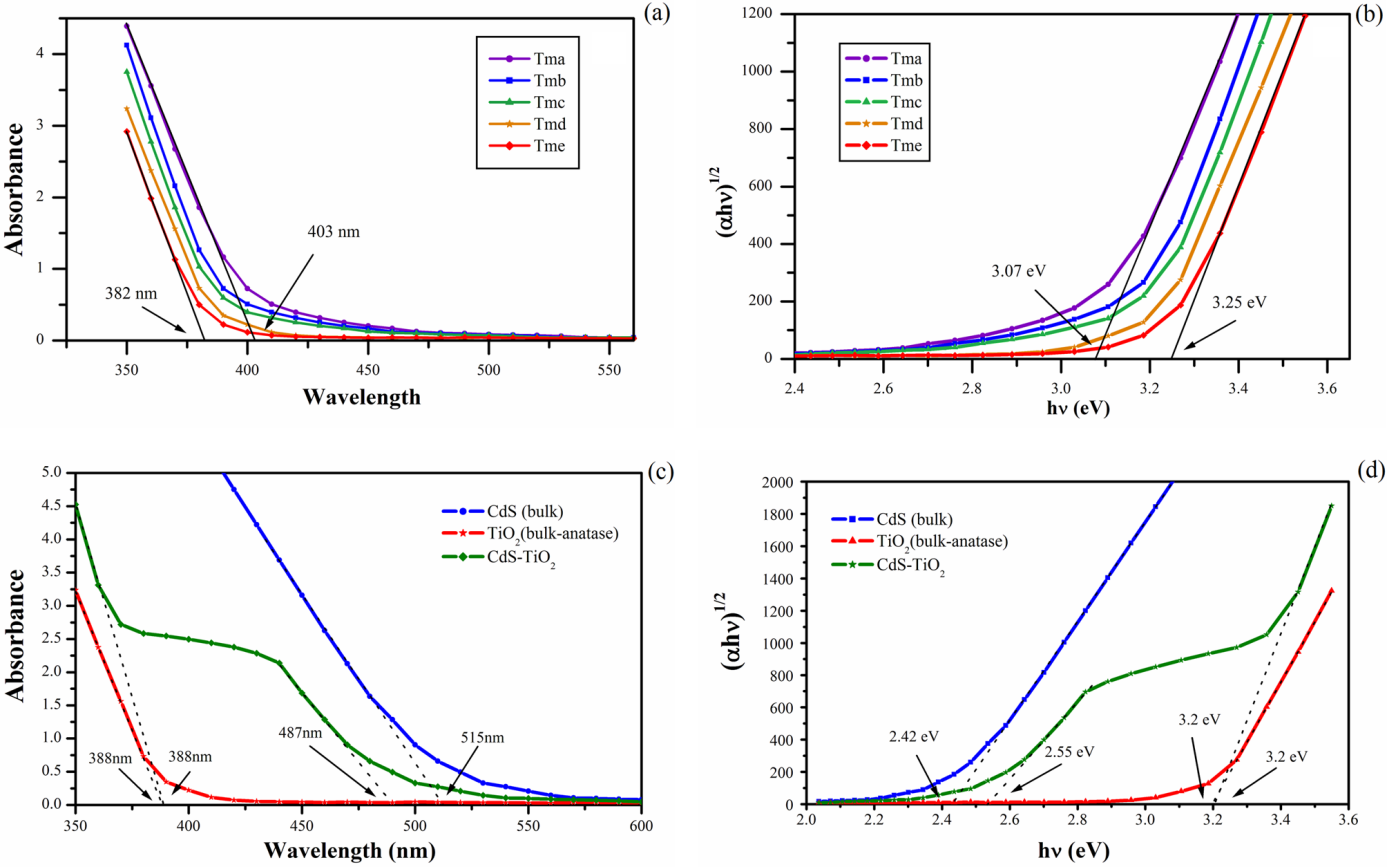
(**a**) The UV–Visible absorption spectrum of synthesized TiO_2_ nanoparticles (Tma, Tmb, Tmc, Tmd and Tme) shows a slight blue shift in absorption edge. (**b**) The bandgap energy of the synthesized TiO_2_ nanoparticles (Tma, Tmb, Tmc, Tmd and Tme). (**c**) The absorption spectra of CdS-TiO_2_ with respect to pure CdS bulk and TiO_2_ bulk. (**d**) Band gap energy curve of CdS-TiO_2_ with respect to pure CdS and TiO_2_ bulk.

The optical properties were determined using the UV–Visible absorption spectra. Figure 9a shows the absorption spectra of TiO_2_ nanoparticles synthesized in the range of 350 nm to 600 nm range of the light spectrum. The absorption edge in bulk TiO_2_ for anatase is 387 nm (3.2 eV), rutile is 410 nm (3.02 eV), and brookite is 381 nm (3.25 eV)^86,87^. The absorption edges of produced TiO_2_ nanoparticles (Tma, Tmb, Tmc, Tmd, and Tme) were found to be in the wavelength range 382–403 nm, implying the presence of mixed phases or a blue shift from the bulk rutile phase. This blue shift was detected with the decrease of TiO_2_ NP particle sizes from Tma to Tme, and was in good agreement with previous findings. This change was linked to QSE or the presence of TiO_2_ mixed-phase^88^. XRD results given in the study indicated the presence of mixed-phase TiO_2_. The values of the absorption edges of the different TiO_2_ NPs are listed in Table 3.

**Table 3:** Absorption peaks and bandgap energy of the synthesized TiO_2_ NP (Tma, Tmb, Tmc, Tmd and Tme) and CdS-TiO_2_.

Nanoparticle	Absorption edge (nm)	Band gap (eV) (from Tauc curve)	Band gap (eV) (E_g_ = 1240/λ_onset_)	Particle size (nm)
Tma	403	3.07	3.074	0.522
Tmb	393	3.14	3.155	0.208
Tmc	390	3.17	3.179	0.188
Tmd	388	3.2	3.195	0.161
Tme	382	3.25	3.246	0.125
CdS-TiO_2_	487, 388	2.55, 3.2	2.546, 3.195	5.8, 0.161

Figure 9b shows the bandgap energy of TiO_2_ NP. The bandgap curve was plotted (αhν)^1/2^ vs hν based on Tauc relation revealed an indirect bandgap^89^. As the size of TiO_2_ NP reduced from Tma to Tme, a greater band gap was observed.

Table 3 lists the corresponding band gaps of the various TiO_2_ NPs. The bandgap of TiO_2_ can also be calculated using the Eq. (1):

1 $${\text{E}}_{{\text{g}}} = 1240/\lambda_{{{\text{onset}}}}$$

where E_g_ denotes the bandgap energy, and onset denotes the absorption edge as determined by the absorption spectra^90^. The results of the above formula were in good agreement with the Tauc relation curves (Table 3).

In Fig. 9c, the absorption spectra of CdS-TiO_2_ are compared to those of bulk CdS and TiO_2_ (anatase). Bulk CdS exhibited a sharp edge at 515 nm, while anatase TiO_2_ exhibited one at 388 nm. The spectrum of CdS-TiO_2_ NC displayed a mixture of these two spectra, with an absorption edge at 487 nm that was blue-shifted from that of pure CdS NP (515 nm) and at 388 nm corresponded to that of pure anatase TiO_2_ NP. The synthesis of anatase TiO_2_ in CdS-TiO_2_ was shown to be good in accordance the XRD results. The creation of a solid solution at the interfaces as a result of close contact between CdS and TiO_2_ caused this shift, and this behaviour determined the optical properties of the final nanostructure^91^. Which attributes to electronic semiconductor-support interaction (SEMSI) by several researchers^92,93^. The UV area is used to excite (bulk) TiO_2_ (bandgap = 3.2 eV), whereas the visible region is used to excite CdS (bandgap 2.42 eV). As a result of visible light absorption, electrons in CdS nanoparticles can be stimulated from the valence band to the conduction band, forming electron–hole pairs that are then trapped by the surface state^94^. These electrons can transfer from the CdS to the TiO_2_ conduction band. They can then migrate across the TiO_2_ conduction band and contribute to the reduction of species like oxygen molecules or adsorbed pollutants^95^. In Fig. 9d, the bandgap curve of CdS-TiO_2_ is compared to bulk CdS and TiO_2_ (anatase) using the Tauc relation. Bulk CdS had a bandgap of 2.42 eV, while bulk TiO_2_ had a bandgap of 3.2 eV. The bandgap curve of CdS-TiO_2_ exhibited a combination of these two, with a bandgap of 3.2 eV for anatase TiO_2_ NP and a bandgap of 2.55 eV for CdS NP.

### Photocatalytic activity

**Figure 10:**
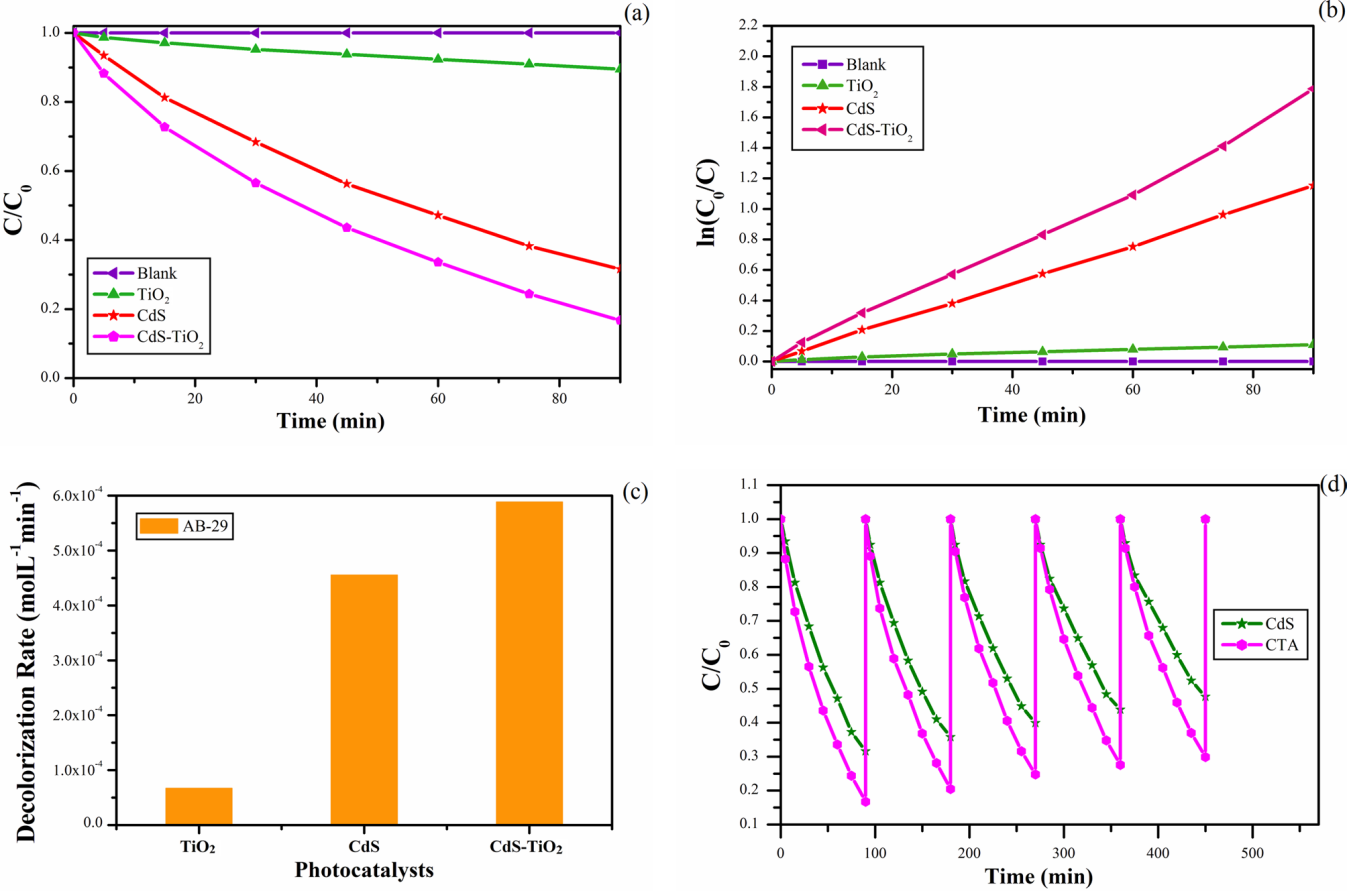
(**a**) Change in concentration of AB-29 with time in the presence and absence of synthesized TiO_2_ nanoparticles (Tma, Tmb, Tmc, Tmd and Tme). (**b**) Change in concentration of AB-29 with time in the presence and absence of synthesized TiO_2_ nanoparticles (Tma, Tmb, Tmc, Tmd and Tme). (**c**) The decolorization rate of AB-29 in the presence of different synthesized TiO_2_ nanoparticles (Tma, Tmb, Tmc, Tmd and Tme). (**d**) Stability and recycle of TiO_2_ nanocomposites (Tma, Tmb, Tmc, Tmd and Tme) for five consecutive cycles.

The effect of samples synthesized in five distinct modes as catalysts on the removal of dye AB-29 was investigated, and the results are shown in Fig. 10a a relative change in AB-29 concentration (C/C_0_) as a function of time in the presence and absence of photocatalysts. The activity of all five samples was compared in order to get superior photocatalytic activities of TiO_2_ nanoparticles. UV light was used to conduct the photo-degradation investigations. Blank experiments were also separately carried out in presence of the photocatalyst under dark conditions and absence of the photocatalyst under irradiation. In both the cases, analysis of the samples did not show any appreciable loss of the dye (AB-29). The percentage of relative decolorization (C/C_0_) of the dye AB-29 was determined in the following order: Tma (71%) Tmb (74%) Tmc (79%) Tmd (83%) Tme (86%). In the absence of photocatalyst, however, there was no discernible decrease in dye concentration. Multiple factors may have contributed to sample Tme's maximum photocatalytic activity.

Most dyes undergo photocatalytic decolorization according to the Langmuir–Hinshelwood kinetic model^96–98^, which can be summarised as (2):

2 $$- {\text{dC}}/{\text{dt}} = {\text{kKC}}/\left( {{1} + {\text{KC}}} \right)$$

where k represents the reaction rate constant (mMmin^−1^), K represents the reactant's adsorption coefficient (mM^−1^), and C represents the reactant concentration (mM). When C is very low, KC is minimal in comparison to unity, allowing Eq. (2) to be simplified to apparent pseudo-first-order kinetics^99^.

3 $$- {\text{dC}}/{\text{dt}} = {\text{kKC}} = {\text{k}}_{{{\text{app}}}} {\text{C}}$$

The decolorization curve (Fig. 10a), emerged as an exponential decay curve which represents pseudo-first-order kinetics reasonably well. The rate constant was obtained for each experiment by plotting the natural logarithm of dye concentration as a function of irradiation time^100^. The following is a representation of the equation:

4 $${\text{ln}}\;\left( {{\text{C}}_{0} /{\text{C}}} \right) = {\text{k}}_{{{\text{app}}}} {\text{t}}$$

C_0_ represents the starting reactant concentration (mM), C represents the reactant concentration (mM) at time “t”, and k_app_ represents the apparent pseudo-first-order rate constant (min^−1^).

The data in Fig. 10a were in good agreement with the pseudo-first-order reaction for our experimental conditions, as shown in Fig. 10b by plotting ln (C_0_/C) versus irradiation time. For all of the experiments, the correlation constant (R^2^) for the fitted lines was calculated to be 0.99.

The dye's degradation rate was estimated using the formula below^101^:

5 $$- \left( {{\text{d}}\left[ {\text{C}} \right]} \right)/{\text{dt}} \to {\text{k}}\left[ {\text{C}} \right]^{{\text{n}}}$$

k = rate constant, C = concentration of the dye, n = order of reaction.

Tme exhibited the maximum activity and nearly fully decolorized the solution in only 90 min, according to the Kinetic findings. The decolorization rate of AB-29 in the presence of several photocatalysts (Tma, Tmb, Tmc, Tmd, and Tme) demonstrated that the decolorization of AB-29 continued faster as the diameters of the TiO_2_ nanoparticles dropped (Fig. 10c). The decolorization rate followed the order; Tma (4.6 × 10^−4^) < Tmb (4.8 × 10^−4^) < Tmc (5.0 × 10^−4^) < Tmd (5.3 xpas 10^−4^) < Tme (5.5 × 10^−4^mol L^−1^ min^−1^). Increased catalyst surface area is responsible for the rise in photocatalytic efficiency when particle size decreases^102^.

**Figure 11:**
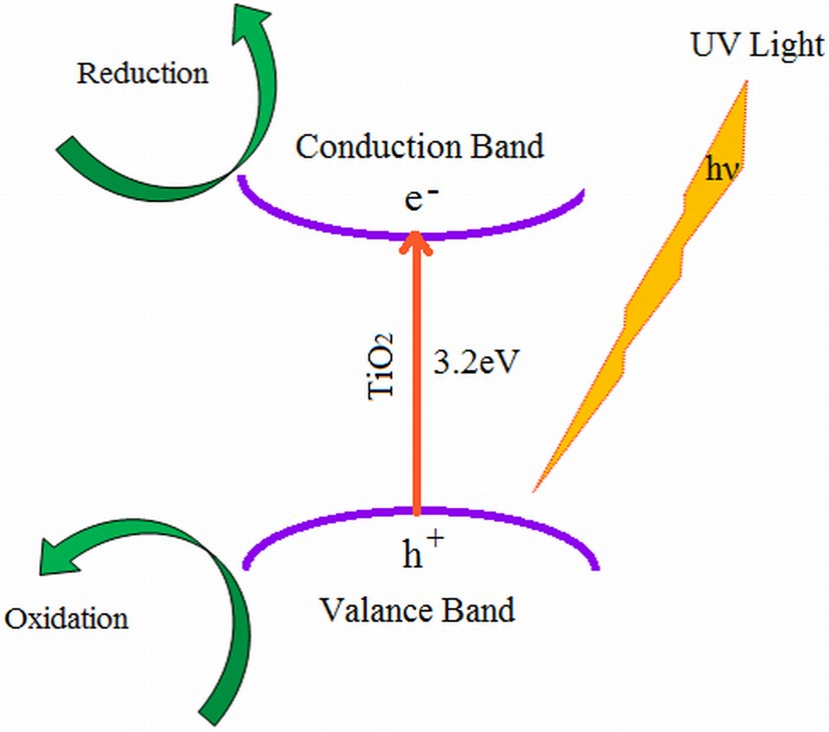
Schematic diagram of the photoexcitation of TiO_2_ under UV light irradiation.

In addition, when particle size decreases, band gap energy rises, reducing charge carrier recombination. It is widely assumed that a bigger band gap equates to greater redox capacity^103^. As a result of its large surface area, enhanced bandgap, and strong redox capability with low photo corrosion, Tme had the highest photocatalytic activity. A photocatalytic reaction using TiO_2_ is essentially a redox reaction including photogeneration, migration, trapping, and recombination of reactants adsorbed on its surface^104^. The process of photocatalysis over titanium dioxide (TiO_2_) can be explained as follows: photocatalysis was initiated by the absorption of a photon with energy equal to or greater than the bandgap of TiO_2_ (3.2 eV), leading to photo-excitation, producing electron–hole (e/h^+^) pairs (Eq. 6)^105^. As a result, the TiO_2_ particle operated as an electron donor or acceptor for molecules in the surrounding medium after irradiation. The photoexcited electron and hole took part in redox reactions with adsorbed species like water, hydroxide ions (OH), organic compounds, and oxygen. The photoexcitation of TiO_2_ under UV light irradiation is depicted in Fig. 11 as an example scheme.

The holes (h^+^) oxidized water (H_2_O) Eq. (7) or hydroxyl anion (OH) Eq. (8) in the valence band to form the hydroxyl radical (·OH), a highly potent and indiscriminate oxidant. Similarly, the electron (e^−^) reduced the adsorbed O_2_ to form superoxide radical anion (O_2_·) Eq. (9) and hydrogen peroxide (H_2_O_2_) Eq. (10), which interacted to produce the ·OH radical Eq. (11). ·OH radicals quickly attacked contaminants on the surface, as well as in solution Eq. (12). This prevented the electron from recombining with the hole and resulted in a concentration of oxygen radical species, which aided in the attack on pollutants^106,107^.

The reactions can be expressed as follows:

6 $${\text{TiO}}_{2} + {\text{h}}\nu \leftrightarrow {\text{TiO}}_{2} ({\text{h}}^{ + } + {\text{e}}^{ - } )$$

7 $${\text{TiO}}_{2} ({\text{h}}^{ + } + {\text{e}}^{ - } ) + {\text{H}}_{2} {\text{O}}_{{{\text{adsorbed}}}} \leftrightarrow {\text{TiO}}_{2} ({\text{e}}^{ - } ) + {\text{H}}^{ + } + {^{ \cdot}}{\text{OH}}$$

8 $${\text{h}}^{ + } + {\text{OH}}_{{{\text{adsorbed}}}}^{ - } \leftrightarrow {^{ \cdot}}{\text{OH}}$$

9 $${\text{e}}^{-}+ {\text{O}}_{2} \to {\text{O}}_{2}^{\cdot -}$$

10 $${2\text{e}}^{- }+ {\text{O}}_{2}+ 2{\text{H}}^{+ }\to {\text{H}}_{2}{\text{O}}_{2}$$

11 $${\text{H}}_{2} {\text{O}}_{2} + {\text{O}}_{2}^{{ \cdot - }} \to {^{ \cdot }}{\text{OH}} + {\text{OH}}^{ - } + {\text{O}}_{2}$$

12 $${^{ \cdot }}{\text{OH}} + {\text{dye}} \to {\text{degradation}}\;{\text{products}}$$

After 90 min of reaction time, the decolorization rates employing TiO_2_ nanocatalysts (Tma, Tmb, Tmc, Tmd, and Tme) for the 5-cycling reuse are shown in Fig. 10d. Table 4 shows the results of five successive cycles of decolorization rates for all TiO_2_ nanocatalysts (Tma, Tmb, Tmc, Tmd, and Tme). The catalytic activity of TiO_2_ nanocatalysts (Tma, Tmb, Tmc, Tmd, and Tme) dropped marginally after the first cycles, according to the findings. Tme showed the most stability among them when compared to other TiO_2_ nanocatalysts, which could be owing to Tme's small size.

**Table 4:** Decolorization rates of all the TiO_2_ nanocatalysts (Tma, Tmb, Tmc, Tmd and Tme) for five consecutive cycles under UV light irradiation.

Cycle	Tma	Tmb	Tmc	Tmd	Tme
I	71.4	74.2	78.6	83.3	85.9
II	70.1	72.7	76.9	80.6	84.8
III	69.0	71.4	74.3	78.5	83.4
IV	67.8	70.4	72.7	76.8	82.1
V	66.7	68.9	71.4	74.4	80.5

Because the differences in relative stability among the TiO_2_ NPs were not significant, it can be concluded that all of the produced TiO_2_ NPs have good photocatalytic activity and UV light irradiation stability. However, because UV radiation accounts for just 4–6% of the total solar spectrum, it is vital to investigate TiO_2_'s applicability in the visible zone.

**Figure 12:**
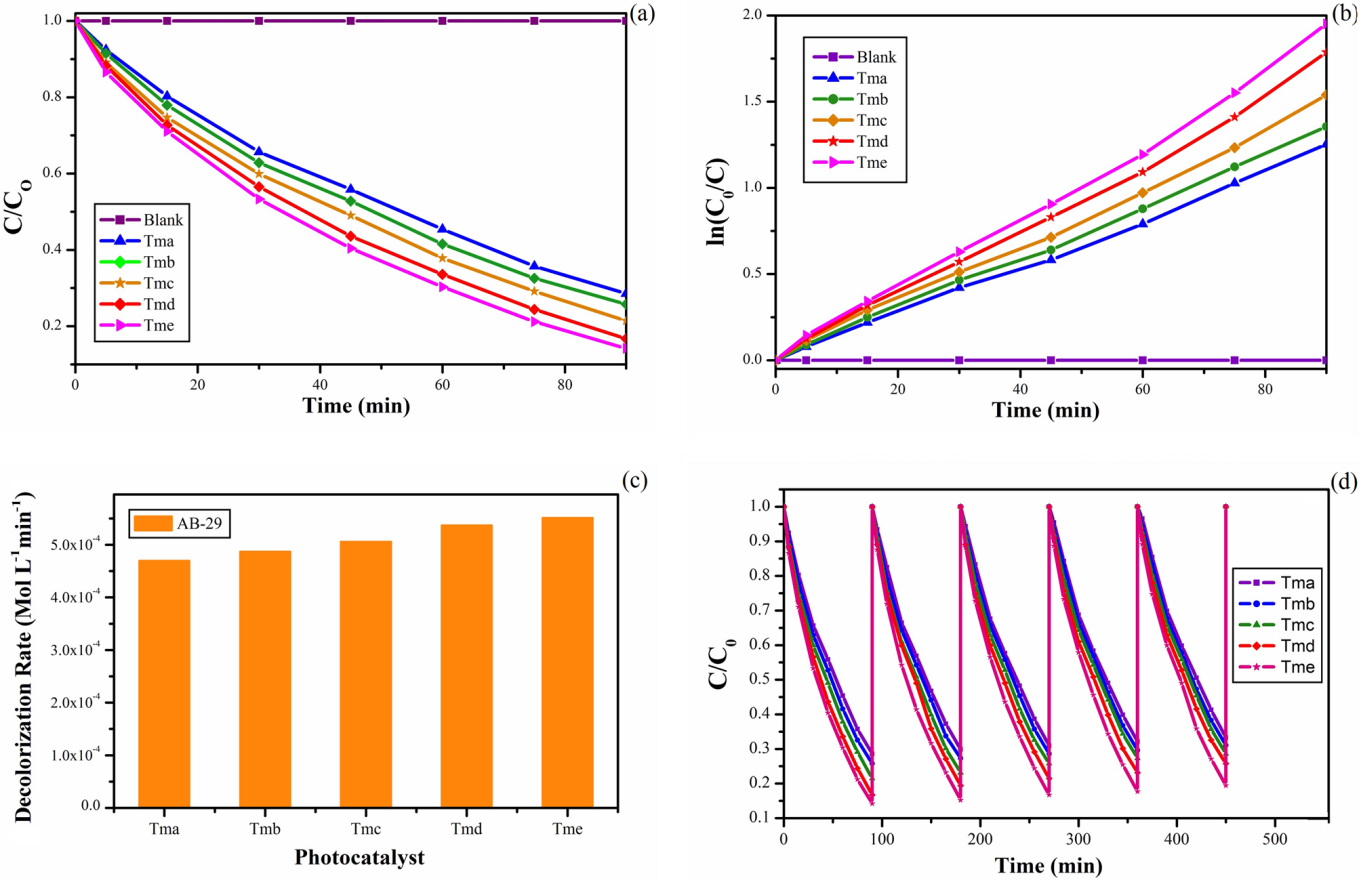
(**a**) Change in concentration of AB-29 with time in the presence and absence of synthesized CdS-TiO_2_ nanoparticles in comparison to pure CdS and TiO_2_ (**b**) Change in concentration of AB-29 with time in the presence and absence of synthesized CdS-TiO_2_ nanoparticles in comparison to pure CdS and TiO_2_. (**c**) The decolorization rate of AB-29 in the presence of different photocatalysts (TiO_2_, CdS and CdS-TiO_2_). (**d**) Stability and recycle of CdS-TiO_2_ nanocomposite in comparison to pure CdS nanocomposite for five consecutive cycles.

As a result, TiO_2_ NPs were combined with CdS NPs (CdS-TiO_2_) and their photoactivity was investigated. A photo-degradation experiment using a dye derivative AB-29 in the presence of visible light was used to investigate the photocatalytic activity of CdS-TiO_2_ nanoparticles. The photocatalytic experiment was conducted in the same manner as the TiO_2_ NP experiment. The absorption edge of CdS-TiO_2_ falls well inside visible radiation, resulting in enhanced photodegradation when exposed to visible light. In the presence of CdS-TiO_2_ nanocomposite and ambient oxygen, irradiation of the dye under examination resulted in the decrease in absorption intensity as a function of irradiation time. In the presence and absence of CdS-TiO_2_ photocatalysts, the relative change in the concentration of AB-29 (C/C_0_) as a function of time is shown in Fig. 12a. The results were compared to nanoparticles of TiO_2_ (Degussa P-25) and CdS^55^. CdS-TiO_2_ demonstrated 84% decolorization of AB-29 after 90 min of visible light irradiation, whereas CdS and TiO_2_ showed only 68% and 09%, respectively, as shown in Fig. 12a. In the absence of photocatalyst, however, there was no discernible decrease in dye concentration. In the visible area, it was confirmed that CdS-TiO_2_ NC had better photocatalytic activity than individual CdS and TiO_2_ NP. The observations were consistent with a pseudo-first-order response, as illustrated in Fig. 12b by plotting ln (C_0_/C) with irradiation time. A visualization of the natural logarithm of dye concentration as a function of irradiation duration Eq. (4) yielded the rate constant. The fitted lines' correlation constant (R^2^) was calculated to be 0.99.

Using the Eq. (5), the dye degradation rate was estimated. The decolorization rate of AB-29 in the presence of CdS-TiO_2_ photocatalyst as shown in Fig. 12c demonstrated that CdS-TiO_2_ decolorized AB-29 was faster (5.8 × 10^−4^ mol L^−1^ min^−1^) than CdS (4.5 × 10^−4^ mol L^−1^ min^−1^) or TiO_2_ (0.67 × 10^−4^ mol L^−1^ min^−1^). The increased photocatalytic effectiveness of CdS-TiO_2_ in the visible area was owing to reduced charge carrier recombination as a result of better charge separation and TiO_2_ extension in response to visible light.

Figure 12d depicts the photodegradation of AB-29 by CdS-TiO_2_ NC and CdS NP over a five-cycle period. After 90 min of response time, the relative decolorization utilising CdS-TiO_2_ for the 5-cycling reuse was 83.3%, 79.6%, 75.4%, 72.4%, and 70.1%, respectively.

The catalytic activity of CdS-TiO_2_ was declined after the first cycles but at a lower rate than that of pure CdS NP, which reduced at a faster rate (68.4%, 64.3%, 60.1%, 56.1% and 52.3% respectively for 5 consecutive cycles). As a result, CdS-TiO_2_ appears to be a superior photocatalyst to pure CdS NP, with increased activity and stability. However, photo corrosion of CdS, which forms cadmium cations, may be the cause of the decrease in CdS-TiO_2_ stability during photocatalytic degradation events.

**Figure 13:**
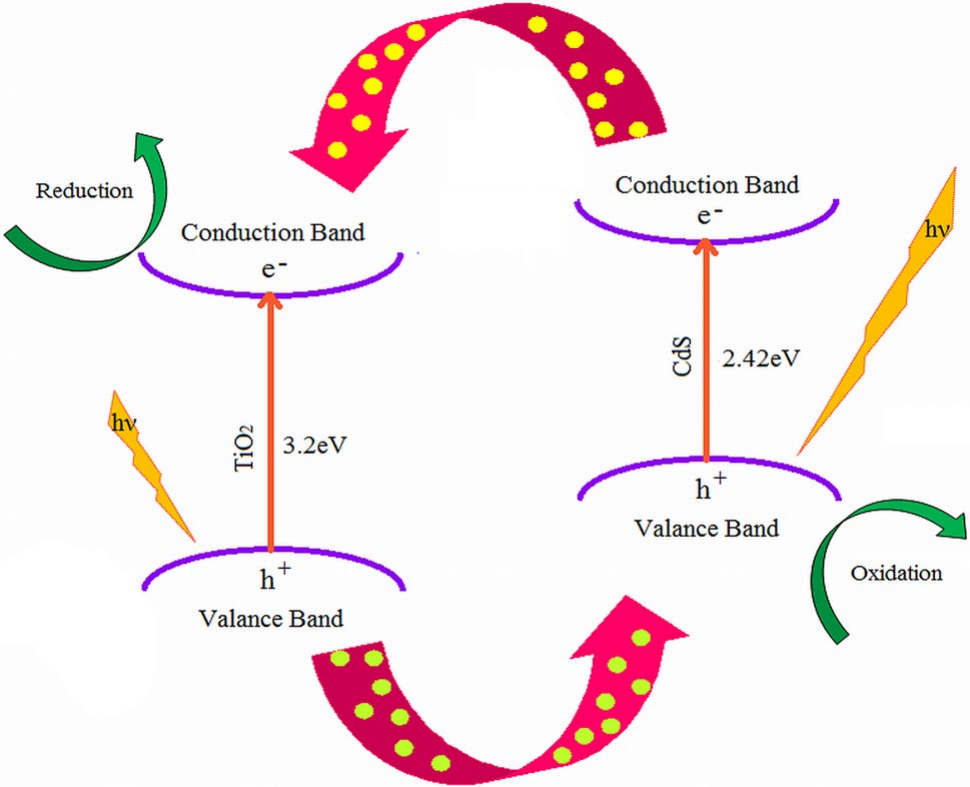
Schematic representation of photocatalytic mechanism followed by CdS-TiO_2_.

Under the instance of CdS/TiO_2_, the narrow band-gap allowed CdS/TiO_2_ to absorb more photons, increasing TiO_2_'s photocatalytic efficiency in the sun. The absorption of a photon by CdS with energy equal to or greater than the bandgap of CdS (2.42 eV) (515 nm) caused excitation of electrons (e^−^) from VB to CB of CdS, leaving a positive vacancy (hole, h^+^) Eq. (13). TiO_2_ also absorbed only a little quantity of visible light, causing photogenerated electrons and holes to occur in the CB and VB of TiO_2_ Eq. (13). Because the conduction band (CB), valence band (VB), and band-gap of the two semiconductors were incompatible and overlapped, photogenerated electrons were directed from the CB of CdS to the CB of TiO_2_, while photogenerated holes were directed from the VB of TiO_2_ to the VB of CdS Eq. (14). Because holes flow in the opposite direction as electrons, they became trapped in the CdS. As a result, charge separation has improved, and recombination has decreased. A proposed mechanism for the degradation of contaminants on CdS coupled TiO_2_ catalyst under visible light irradiation is given in Fig. 13 based on literature findings^13,19,79,89,91,92^ and our experiment results.

In oxygen-equilibrated environments, the photoexcited and transmitted electrons in TiO_2_'s CB were scavenged by molecular oxygen O_2_, yielding the superoxide radical anion O_2_· Eq. (15) and hydrogen peroxide H_2_O_2_ Eq. (16). The interaction of these intermediates resulted in the formation of the hydroxyl radical ·OH Eq. (17). The oxidative breakdown of AB-29 eq was then triggered by the ·OH radical Eq. (18)^91^. The photo-generated and transmitted hole in the VB of CdS cannot form hydroxyl radicals by oxidizing hydroxyl groups and H_2_O molecules, but it can oxidize dye molecules to reactive intermediates and then to final products Eq. (19).

The reactions can be expressed as follows:

13 $${\text{CdS}}/{\text{TiO}}_{2} \mathop \to \limits^{{{\text{h}}\nu }} {\text{CdS}}\left( {{\text{e}}^{ - } + {\text{h}}^{ + } } \right)/{\text{TiO}}_{2} \left( {{\text{e}}^{ - } + {\text{h}}^{ + } } \right)$$

14 $$\text{CdS}\left({\text{e}}^{-}+{\text{h}}^{+}\right)/{\text{TiO}}_{2}\left({\text{e}}^{-}+{\text{h}}^{+}\right)\to \text{CdS}\left({\text{h}}^{+}\right)/{\text{TiO}}_{2}\left({\text{e}}^{-}\right)$$

15 $$\text{CdS}\left({\text{h}}^{+}\right)/{\text{TiO}}_{2}\left({\text{e}}^{-}\right)+ {\text{O}}_{2} \to \text{ CdS}\left({\text{h}}^{+}\right)/{\text{TiO}}_{2}+{\text{O}}_{2}^{\cdot -}$$

16 $$\text{CdS}\left({\text{h}}^{+}\right)/{\text{TiO}}_{2}\left({\text{e}}^{-}\right)+ {\text{O}}_{2}+ 2{\text{H}}^{+ }\to \text{ CdS}\left({\text{h}}^{+}\right)/{\text{TiO}}_{2}+{\text{H}}_{2}{\text{O}}_{2}$$

17 $${\text{H}}_{2} {\text{O}}_{2} + {\text{O}}_{2}^{{ \cdot - }} \to {^{ \cdot }}{\text{OH}} + {\text{OH}}^{ - } + {\text{O}}_{2}$$

18 $${^{ \cdot}} {\text{OH}} + {\text{dye}} \to {\text{degradation}}\;{\text{products}}$$

19 $${\text{CdS}}\left( {{\text{h}}^{ + } } \right)/{\text{TiO}}_{2} + {\text{Dye}} \to {\text{CdS}}/{\text{TiO}}_{2} + {\text{Dye}}^{{ \cdot + }} \to {\text{CdS}}/{\text{TiO}}_{2} + {\text{degradation}}\;{\text{product}}$$

Because photo-generated holes in CdS nanocrystals are unable to convert hydroxyl groups to hydroxyl radicals due to their valence band potential, photo corrosion of CdS occurs, resulting in the formation of cadmium cations^108–111^. The decrease in photo-stability of CdS-TiO_2_ in the recycle experiment (Fig. 12d) also indicated leaching of cadmium cations. Table 5 provides additional examples of reports for the degradation performance of CdS and other nanocomposites for AB-29 azo dye as organic pollutants at the indicated experimental conditions.

**Table 5:** Comparative degradation of AB-29 azo dye by various nanocomposites.

Catalyst	D (%)	Refs
Montmorillonite K10-Cu(II)ethylenediamine (MMTK10-Cu(en)2)	90	^112^
CdS	98	^60^
CdS with Sulphide ions	98	^113^
Fe (II) doped CdS	98.75	^114^
ZnFe_2_O_4_	98.83	^115^
CdS-TiO_2_	84	Present work

## Conclusion

Under ambient conditions, Titanium Dioxide nanoparticles (Tma, Tmb, Tmc, Tmd, and Tme) were effectively produced using a single pot chemical precipitation approach. The size of the TiO_2_ nanocluster was reduced as the concentration of the Ti precursor dropped, as seen by the prepared TiO_2_ NP. In all TiO_2_ samples, a mixed crystalline phase was detected. The micrographic analysis demonstrated the production of spherical clusters whose diameters shrank drastically as the Ti precursor concentration dropped. With the decrease in Ti precursor concentration, the absorption spectra indicated a minor blue shift and, as a result, a slight rise in bandgap energies of TiO_2_ NP was observed. Finally, under UV light, the photocatalytic response for the breakdown of the organic dye AB-29 was improved as TiO_2_ had photoactive under UV light and CdS found as photocorroded during photocatalytic processes, an effort was made to combine the beneficial qualities of both CdS and TiO_2_ while minimizing their disadvantages. A sandwich-type nanocomposite (CdS-TiO_2_) of CdS with TiO_2_ was also successfully synthesized. The CdS-TiO_2_ showed good elemental purity and thermal stability. Cubic CdS and anatase TiO_2_ were discovered in the XRD spectrum. Because of the presence of TiO_2_, the absorption edge of CdS in CdS-TiO_2_ shifted slightly blue, but the absorption edge of TiO_2_ was identical to that of pure anatase TiO_2_, indicating an improvement in the crystal structure. TiO_2_'s optical sensitivity was moved towards the visible range in CdS-TiO_2_, allowing it to be photocatalytically active in visible light as well. With the decrease in Ti precursor concentration, the absorption spectra indicated a minor blue shift and, as a result, a slight rise in bandgap energies of TiO_2_ NP.
